# Amniotic Fluid Embolism Treated with Veno-Arterial Extracorporeal Membrane Oxygenation

**DOI:** 10.1155/2019/4589636

**Published:** 2019-12-21

**Authors:** Robert Gitman, Bradlee Bachar, Byron Mendenhall

**Affiliations:** Kaweah Delta Medical Center, Visalia, CA, USA

## Abstract

Amniotic fluid embolism (AFE) is an extremely rare yet fatal obstetric emergency. AFE presents as sudden cardiovascular collapse after a breach of maternal-fetal membranes and is often complicated by severe coagulopathy. We present a case where an AFE was treated with veno-arterial extracorporeal membrane oxygenation (ECMO) to help overcome the acute cardiopulmonary insult. The use of echocardiography proved to be an invaluable tool to help guide treatment and optimal duration of ECMO in the face of severe coagulopathy.

## 1. Introduction

AFE often presents as immediate and severe hemodynamic collapse after delivery of the infant. Approximate incidence rates are 1/40000 deliveries with case fatality and perinatal mortality rates between 11–80% and 7–44%, respectively [1]. The Society of Maternal Fetal Medicine's treatment recommendations focus on the importance of resuscitation and supportive care in the acute setting [2]. However, high mortality rates demonstrate that traditional measures often do not provide enough cardiopulmonary support to overcome the severity of this disease process. We herein present a unique case of a patient with a clinical AFE whose critical condition progressed to cardiopulmonary collapse and disseminated intravascular coagulopathy (DIC) for which veno-arterial ECMO was required as a life-saving intervention.

## 2. Case Report

A 42-year-old nulliparous woman with no significant past medical history presented to the hospital at 34 2/7 weeks gestation with pre-eclampsia with severe features (BP 205/112 mmHg, 3+ proteinuria). Initially asymptomatic, the obstetric evaluation demonstrated normal fetal movement with no fetal distress and the patient was admitted for magnesium sulfate and steroid therapy. After twenty-four hours, she was considered noninducible due to multiple uterine fibroids, thus cesarean section was performed. Spinal anesthetic (hyperbaric 0.75% Bupivacaine 15 mg, Fentanyl 15 mcg, preservative free Morphine 0.2 mg) was uncomplicated and achieved a T6 level of anesthetic. Cesarean section was performed via a low transverse incision with clear amniotic fluid. Due to large uterine fibroids, external pressure was placed on the uterus to position the neonate for vacuum suction delivery. During delayed cord clamping, the mother became unresponsive, hypoxic, hypotensive (NIBP 48/25 mmHg) and was promptly intubated. The patient further decompensated to PEA arrest and ACLS protocol was initiated. After multiple rounds of CPR and epinephrine administration, return of spontaneous circulation (ROSC) was achieved. Central venous and radial artery catheters were placed while the patient was placed on norepinephrine and vasopressin infusions to maintain blood pressure. Intraoperative transthoracic echocardiogram (TTE) demonstrated right ventricular dilatation with a large pericardial effusion. The clinical setting of rapid hemodynamic collapse after cesarean section raised serious concerns for an amniotic fluid embolism. Atropine 1 mg, ondansetron 4 mg, and ketorolac 30 mg (A-OK) were administered to treat the AFE, as described by Rezai et al., but the patient remained in cardiovascular shock [3]. The placenta was then delivered without difficulty and the uterus cavity wiped with lap sponges but noted to be difficult due to large fibroids. Due to residual blood in uterus, a JP drain was left in place and rectal misoprostol was administered.

**Figure 1:**
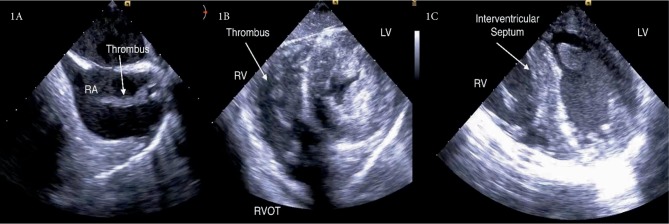
Intraoperative TEE before veno-arterial-ECMO cannulation. (A) Bicaval view-large thrombus visualized in the right atrium (RA) from static blood flow and coagulopathy. (B) Trans-gastric SAX-Right ventricle (RV) thrombus occluding the right ventricular outflow tract (RVOT). Left ventricle (LV) is hyperdynamic and underfilled. (C) Four chamber view-septal bowing into the LV, D-shaped septum suggesting RV pressure overload.

The patient was emergently transferred to the cardiovascular operating room for veno-arterial ECMO cannulation, pericardial window, followed by hysterectomy for uncontrollable uterine bleeding. Intraoperative transesophageal echocardiogram (TEE) showed a large thrombus in the right atrial and ventricular chambers obstructing both the tricuspid valve and right ventricular outflow tract. The right ventricle appeared dilated and dyskinetic while the left ventricle was underfilled and hyperdynamic (Figure 1).

Upon admission to the cardiovascular intensive care unit (CVICU), the patient was in fulminant DIC (D-dimer level >5250 *µ*g/mL, fibrinogen 35 mg/dl, platelets 50, INR >10 and PTT >200 seconds). Hours after arriving in the CVICU, the patient had increasing abdominal distention which raised concern for abdominal compartment syndrome. Exploratory laparotomy revealed intra-abdominal hemorrhage from a left fallopian tube bleed with the evacuation of >3000 cc of blood. Over the next 22 hours, DIC persisted requiring multiple transfusions of packed red blood cells (pRBC), fresh frozen plasma (FFP), platelets, and cryoprecipitate as well as two additional abdominal washout procedures for intractable intra-abdominal hemorrhage (2000 cc, 1000 cc blood loss respectively). Overall blood products totaled 93 units pRBC, 12 units of platelets, 59 units of FFP, and 9 units of cryoprecipitate.

**Figure 2:**
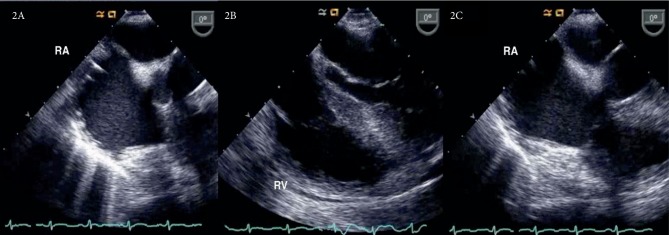
Intraoperative TEE before veno-arterial ECMO decannulation. (A) Right atrial view-RA shows resolution of thrombus load. (B) Four chamber view-normalization of RV/LV ratio. Resolution of septal bowing. (C) Four chamber view-RA dilation persisted despite overall clinical improvement and resolution of thrombus load.

Although veno-arterial ECMO provided support for an acutely dysfunctional cardiopulmonary system, the circuit was thought to be damaging necessary clotting factors and causing hemolysis; worsening the consumptive coagulopathy. Serial bedside TEE exams showed decreased thrombus load, resolution of RV dilatation, and progressive improvement in contractility (Figure 2). Thus, the patient was decannulated from veno-arterial ECMO 26 hours after initiation.

Two days after decannulation, the patient was titrated off inotropic support and was able to maintain a MAP greater than 65 mmHg. Four days after decannulation, the patient was successfully weaned off the ventilator and extubated. Over the next 2 weeks, the patient regained baseline neurological function and was discharged to a rehabilitation center after a 25-day hospitalization.

## 3. Discussion

AFE was initially described by Meyer in 1926 [4]. In 1941, Steiner and Lushbaugh proposed the etiology of a physical obstruction of maternal pulmonary circulation by fetal amniotic material [5]. Over the years, inconsistencies in laboratory evidence and autopsy findings were unable to validate this theory. In 1995, Clark et al. demonstrated that the etiology of AFE was immunologically mediated by activation of the complement cascade and mast cell degranulation; coining the term “Anaphylactoid Syndrome of Pregnancy” [6].

Clark et al. proposed a biphasic mode of the hemodynamic consequences of AFE. The initial response is an acute pulmonary vasoconstriction, hypoxia, and right heart failure [7]. The right heart failure leads to septal bowing into the left ventricle (D-sign), decreasing cardiac output, worsening cardiogenic shock and pulmonary edema. If one survives the initial insult, the pulmonary vasoconstriction is generally not sustained and improves within a few hours to days. AFE requires an influx of fetal components (platelet activating factor, phospholipase A2, thromboplastin, endothelin and prostaglandins) which have vasoactive and pro-coagulant properties. The late phase of the disease process is mediated by tissue factor found in amniotic fluid, which binds to Factor VII to activate the extrinsic coagulation cascade leading to DIC and severe hemorrhage [8].

The most common presenting signs and symptoms include hypotension (100%), hypoxia, altered mental status/encephalopathy, tonic-clonic seizures (10–50%), uterine atony, and DIC (83%) [8]. Four criteria must be present for diagnosis of AFE: (1) acute hypotension or cardiac arrest, (2) acute hypoxia, (3) coagulopathy or severe hemorrhage in the absence of other explanations, (4) all of these occurring during labor, cesarean delivery, dilation and evacuation, or within 30 minutes postpartum with no other explanation of findings [8].

Initial diagnostic evaluation should include labs which aid in resuscitation such as arterial blood gas (ABG), complete blood count (CBC), complete metabolic panel (CMP), prothrombin time /thromboplastin time (PT/PTT), blood type/screen, fibrinogen, and thromboelastogram. The anaphylactoid nature of the disease process has led many to consider laboratory or pathological evidence to objectively diagnose the condition. There have been reports of elevated levels of serum tryptase and urine histamine but neither have been found to be sensitive serological markers [9]. The Society of Maternal Fetal Medicine does not recommend any laboratory test to either confirm or refute the diagnosis of AFE [2]. Diagnostic imaging may be challenging in the setting of acute hemodynamic instability, thus bedside echocardiography has been found to be an invaluable tool in this setting. In our case, the initial TTE clearly demonstrated a dyskinetic under-filled left ventricle and right heart strain which directly led to the decision for veno-arterial ECMO. Additionally, serial TEE exams showed improved cardiac function and decreased thrombus load which helped providers decide the optimal time for ECMO decannulation.

To optimize treatment in the acutely unstable setting, a wide differential including must be kept including all types of shock: anaphylactic, hemorrhagic, septic, and obstructive due to a pulmonary embolism or air embolism. Of note, the cardiopulmonary collapse caused by a large pulmonary embolism may present similarly but is not associated with the coagulopathy or timing seen in an AFE. It is recommended that management be focused on resuscitation via intravascular volume repletion, correcting coagulopathy with appropriate blood products, controlling hemorrhage, administering inotropic and vasopressor support, as well as methods to prevent multi-organ dysfunction such as renal replacement therapy (RRT) [8]. Ihara et al. suggested that RRT may filter fetal remnants of amniotic fluid from maternal circulation, thus provide renal protection and prevention of disease progression [10]. Additionally, other novel approaches have successfully been shown to treat AFE, including plasmapheresis, ventricular assist devices, intra-aortic balloon pump, and ECMO [1].

The development and advancements in ECMO over recent years has led it to be considered a viable treatment modality for high risk cardio-pulmonary failure. This is especially true in patients who have developed instability from an acute event and do not have multiple other chronic illnesses/co-morbidities. Thus, early usage of veno-arterial ECMO may decrease cardiac work and adequately oxygenate the patient until the transient acute anaphylactoid phase of the disease process is overcome.

In 2015, the extracorporeal life support registry reported a 57% mortality rate of adults who have undergone ECMO for cardiogenic shock with worse outcomes associated with prolonged cannulation [11]. Major complications should be considered with ECMO, most notably coagulopathy. The ECMO circuit is believed to cause a consumptive coagulopathy by shear stress and turbulent flow. The circuit causes hemolysis and consumes necessary platelets, clotting factors, and Von Willebrand factor [12]. The coagulopathy associated with AFE in combination with ECMO makes hemorrhage a likely cause of morbidity. Thus, timely decannulation is key to optimizing patient care. To assess the status of the patient's heart, serial bedside TEE's were utilized to trend cardiac function and thrombus load. When the TEE showed improvement, the patient was urgently decannulated with the goal of improving the severe coagulopathy. Within hours after decannulation, it was apparent that the hemorrhage subsided and labs trended favorably; subsequent abdominal washout procedures had a total of 1000 cc blood loss, compared to the 6000 cc while on ECMO. Case reports describing the use of veno-arterial ECMO to treat AFE vary greatly in presentation and there is no consensus on optimal duration of cannulation. In the case presented, the patient was supported by veno-arterial ECMO for a total of 26 hours.

## 4. Conclusion

An amniotic fluid embolism is a rare and often fatal syndrome with extensive case by case variability. Initial treatment strategy requires a multidisciplinary approach which focuses on early recognition and resuscitation. In this case, early veno-arterial ECMO cannulation to optimize support for an acutely dysfunctional cardiopulmonary system was found to be beneficial. Although balancing DIC in combination with ECMO proved to be extremely challenging, use of serial bedside TEE was an invaluable tool to assess cardiac function and optimal time for decannulation. Early ECMO decannulation was paramount to improving hematological dyscrasias and eventual survival.
